# The complete chloroplast genome sequence of *Arundo formosana* Hack. (Poaceae)

**DOI:** 10.1080/23802359.2021.1972865

**Published:** 2021-08-31

**Authors:** Li-Ying Feng, Chao Shi, Li-Zhi Gao

**Affiliations:** a Institution of Genomics and Bioinformatics, South China Agricultural University, Guangzhou, China; b Plant Germplasm and Genomics Center, Kunming Institute of Botany, Chinese Academy of Sciences, Kunming, China

**Keywords:** *Arundo formosana*, chloroplast genome, Poaceae, phylogeny

## Abstract

*Arundo formosana* Hack. belongs to the Arundionideae subfamily of Poaceae. In this study, we sequenced and assembled the complete chloroplast genome of *A. formosana.* The complete chloroplast genome was 136,919 bp in size, including a large single copy region of 82,039 bp, a small single-copy region of 12,108 bp and a pair of reverse repeats of 21,386 bp in size. The annotation of *A. formosana* indicates that it contained 81 protein-coding genes, 47 tRNA and 8 rRNA. Our phylogenetic analysis of the 36 grass complete chroloplast genomes of protein-coding genes using *Cyperus rotundus* as outgroup showed that *A. formosana* is closely related to *Crinipes* species to form the Arundionideae lineage of the grass family.

*Arundo formosana* Hack. belongs to the Arundionideae subfamily of Poaceae. It is a kind of grass growing on the edge of the coastal rock wall or the grassland on the hillside, 350–450 meters above sea level. The species is endemic in Taiwan and Yunnan provinces of China. Based on five intergenic regions of chloroplast DNA fragments, a phylogeographic study of *A. formosana* demonstrates the pertinence of infraspecific taxa in integrative taxonomy and phylogeography below the species level (Hardion et al. 2017). Indeed, subspecies, varieties, and morphologies are the most common taxonomic levels under the species level (McNeil et al. 2012). Recent decades have witnessed extensive efforts to reconstruct the phylogeny of Gramineae. However, the phylogenetic relationships within several clades have not been fully resolved (Clark et al. 1995). The analysis of 81 genes from 64 plastid genomes have fully resolved relationships in angiosperms and identified genome-scale evolutionary patterns (Jansen et al. 2007). The chloroplast DNAs (cpDNAs) sequences have been increasingly used for resolving the deep phylogeny of plants because of their low rates of nucleotide substitutions and genomic structural changes (Jansen et al. 2007). Thus, there is a still great demand to generate the complete chloroplast grass genomes to question about origins and evolution of Poaceae plants on Earth.

In this study, *A. formosana* plants were collected in the suburbs of Menghai (22°28′32″N, 99°56′30″E), Xishuangbanna State, Yunnan Province, China. The voucher specimen (SCAU 2020148) was deposited in SCAU (the herbarium of the College of Agriculture, South China Agricultural University https://nxy.scau.edu.cn, Li-zhi Gao, SCAUgenomics@163.com), China. About 20 g fresh mature leaves were sampled from *A. formosana*, and cpDNAs were extracted by following a modified high salt method reported formerly (Shi et al. 2012). After the cpDNA isolation, approximately 5–10 µg of DNA was sheared, followed by adapter ligation and library amplification, and then subjected to Illumina Sample Preparation Instructions. The fragmented cpDNAs were sequenced at both single-read using the Illumina Genome Analyzer IIx platform at the in-house facility at The Germplasm Bank of Wild Species in Southwestern China, Kunming, China. The obtained paired-end reads (2 × 100 bp read lengths) were assembled using SOAP *de novo* (Li et al. 2010). Regions with ambiguous alignment (conflicted reads mapped to the same genomic region) were trimmed off manually and considered as gaps. Polymerase chain reaction (PCR) amplified fragments yielded by primers derived from the terminal ends of contigs, and the fragments were then sequenced to flank the gap regions. The PCR amplification reactions were template denaturation at 80 °C for 5 min followed by 30 cycles of denaturation at 95 °C for 30 sec, primer annealing at 55 °C for 30 sec, and primer extension at 65 °C for 1 min; followed by a final extension step of 5 min at 65 °C. PCR products were separated by electrophoresis in 1.5% agarose gel and sequenced on an Applied Biosystems (ABI) 3730 automated sequencer. Subsequently, gene prediction and annotation were performed by DOGMA (Wyman et al. 2004).

The complete chloroplast genome of *A. formosana* was 136,919 bp in size, comprising two inverted repeat regions (IRs) with a total of 42,772 bp in size, which were split by a large single copy (LSC) with 82,039 bp and small single copy (SSC) with 12,108 bp in length. The chloroplast genome contained 136 functional genes, including 81 protein-coding genes, 47 tRNAs, and 8 rRNAs. A total of 16 genes were repeated in the IR regions, including 4 rRNA genes (*rrn16*, *rrn23*, *rrn4.5*, and *rrn5*), 6 protein-coding genes (*rpl23*, *ycf15*, *ndhB*, *rps15*, *rps12* and *rps7*) and 6 tRNA genes (*trnI-CAU*, *trnL-CAA*, *trnV-GAU*, *trnI-GAU*, *trnR-ACG*, and *trnN-GUU*). The overall GC content of the *A. formosana* chloroplast genome was ∼38.73% with the corresponding values of 36.87%, 33.12% and 44.93% in the LSC, SSC, and IR regions, respectively.

To determine the phylogenetic position of *A. formosana* in the grass family, 35 grass chloroplast genomes of all protein-coding genes together with *Cyperus rotundus* from Cyperaceae were separately downloaded from GenBank. Phylogenomic analysis was performed by incorporating the *A. formosana* chloroplast genome obtained in this study. All sequences were aligned with MAFFT 7.409 (Katoh et al. 2002). Using *C. rotundus* as outgroup phylogenetic tree was reconstructed using the maximum likelihood method using RAxML (Stamatakis 2014) based on 1,000 bootstrap replicates. Our results indicated that the 35 examined grass species were evidently clustered into the twelve subfamilies of Poaceae with strong bootstrap supports (Figure 1). It is apparent that *A. formosana* is closely related to *Crinipes* species from Arundionideae of the grass family with strong bootstrap supports.

**Figure 1.**
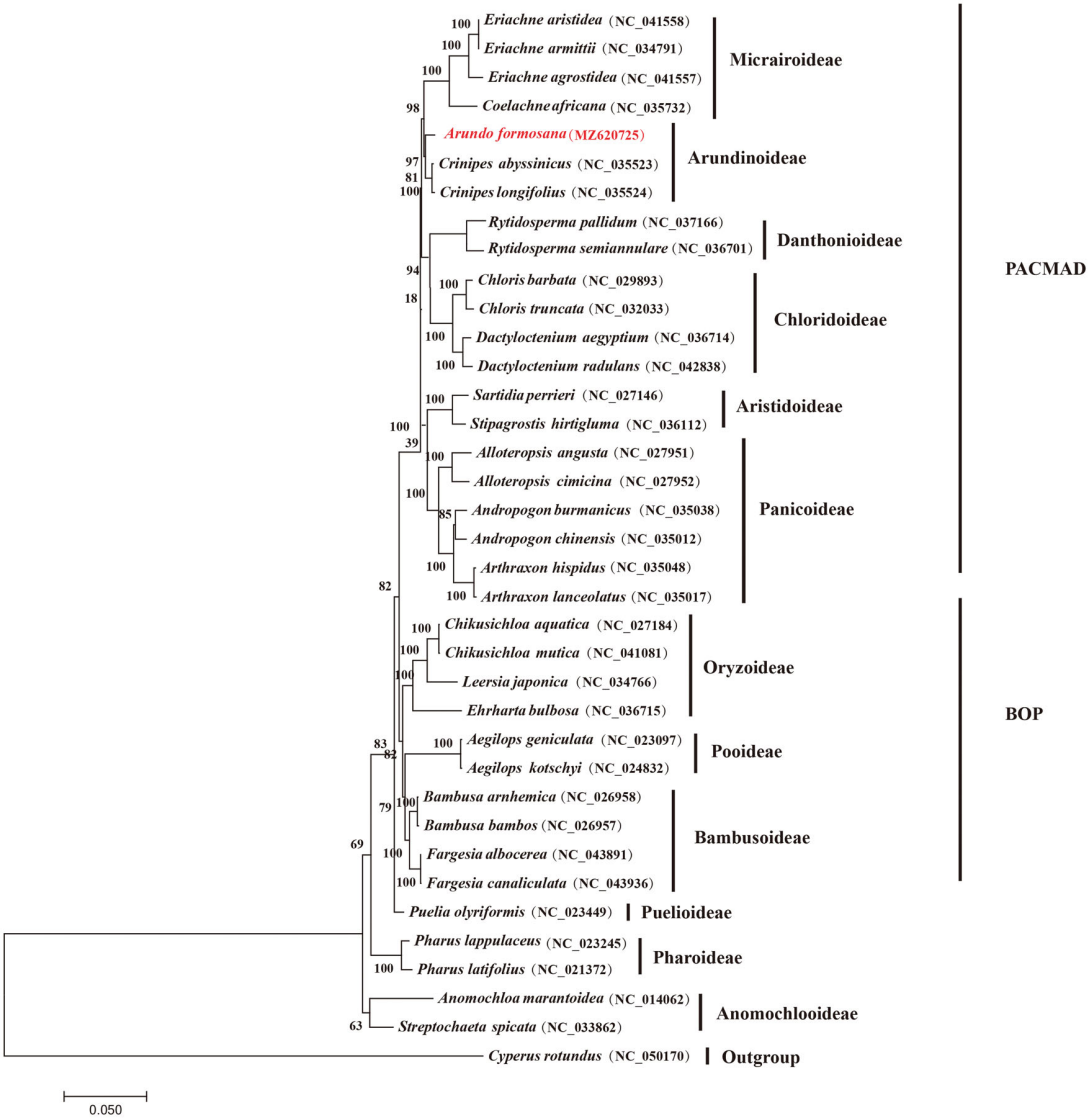
Maximum likelihood phylogenetic tree based on all protein-coding genes of the 36 grass complete chloroplast genomes using *Cyperus rotundus* as outgroup. Bootstraps values (1000 replicates) are shown at the nodes.

## Data Availability

The genome sequence data that support the findings of this study are openly available in GenBank of NCBI at [https://www.ncbi.nlm.nih.gov] (https://www.ncbi.nlm.nih.gov/) under the accession no. MZ620725. The associated BioProject, SRA, and Bio-Sample numbers are PRJNA744348, SRR15130727, and SAMN20166948 respectively. The data that newly obtained at this study are also publicly available in the National Genomics Data Center at https://ngdc.cncb.ac.cn under the accession number of GWHBCHV00000000.
